# Electrophysiological cardiovascular MR: procedure-ready mesh model generation for interventional guidance based on non-selective excitation compressed sensing whole heart imaging

**DOI:** 10.1038/s41598-024-59230-0

**Published:** 2024-04-18

**Authors:** Cosima Jahnke, Angeliki Darma, Frank Lindemann, Sabrina Oebel, Sebastian Hilbert, Kerstin Bode, Christian Stehning, Jouke Smink, Ingo Paetsch

**Affiliations:** 1 Department of Electrophysiology, HELIOS Heart Center Leipzig at University of Leipzig, Struempellstr. 39, 04289 Leipzig, Germany; 2 Present Address: Philips Research Laboratories, Best, The Netherlands; 3 Philips Research Laboratories, Hamburg, Germany

**Keywords:** Cardiovascular magnetic resonance imaging, CMR-guided intervention, EP-CMR, Whole heart imaging, Non-selective SSFP, Compressed SENSE, Isthmus-dependent atrial flutter, Radiofrequency ablation, Interventional cardiology, Cardiology

## Abstract

Fully CMR-guided electrophysiological interventions (EP-CMR) have recently been introduced but data on the optimal CMR imaging protocol are scarce. This study determined the clinical utility of 3D non-selective whole heart steady-state free precession imaging using compressed SENSE (nsWHcs) for automatic segmentation of cardiac cavities as the basis for targeted catheter navigation during EP-CMR cavo-tricuspid isthmus ablation. Fourty-two consecutive patients with isthmus-dependent right atrial flutter underwent EP-CMR radiofrequency ablations. nsWHcs succeeded in all patients (nominal scan duration, 98 ± 10 s); automatic segmentation/generation of surface meshes of right-sided cavities exhibited short computation times (16 ± 3 s) with correct delineation of right atrium, right ventricle, tricuspid annulus and coronary sinus ostium in 100%, 100%, 100% and 95%, respectively. Point-by-point ablation adhered to the predefined isthmus line in 62% of patients (26/42); activation mapping confirmed complete bidirectional isthmus block (conduction time difference, 136 ± 28 ms). nsWHcs ensured automatic and reliable 3D segmentation of targeted endoluminal cavities, multiplanar reformatting and image fusion (e.g. activation time measurements) and represented the basis for precise real-time active catheter navigation during EP-CMR ablations of isthmus-dependent right atrial flutter. Hence, nsWHcs can be considered a key component in order to advance EP-CMR towards the ultimate goal of targeted substrate-based ablation procedures.

## Introduction

Cardiovascular magnetic resonance (CMR) imaging excels in providing detailed three-dimensional anatomical information together with excellent soft tissue contrast. With regard to arrhythmia patients, the unique ability for pre-interventional myocardial substrate delineation and post-interventional lesion characterization plays a pivotal role^1,2^. Consequently, CMR imaging has already become a valuable tool for diagnostic assessment, electrophysiological procedure (EP) planning, and therapeutical stratification of patients with atrial or ventricular rhythm disorders. To further exploit the inherent advantages of CMR imaging, fully CMR-guided ablation procedures (EP-CMR) are reasonably considered the ultimate goal.

More recently, cavo-tricuspid isthmus ablation has been successfully introduced as the first EP-CMR application ready for clinical routine usage^3,4^. Cavo-tricuspid isthmus ablation represents a strictly anatomically defined electrophysiological intervention and was, thus, ideally suited for the development and implementation of an initial EP-CMR set-up^3,5^. The accurate and rapidly available three-dimensional anatomical visualization of all cardiac and vascular cavities was identified as an indispensable prerequisite for precise catheter navigation to the targeted ablation site during the EP-CMR procedure. At present, whole heart imaging using navigator-gated 3D steady-state free precession (SSFP) represents an acknowledged standard but requires relatively long scan durations and may result in degraded image quality due to inhomogeneous endoluminal signal intensity distribution leading to impaired or prolonged image dataset segmentation^6^. The recent introduction of compressed SENSE for cardiac imaging together with a non-selective radiofrequency excitation for SSFP imaging holds the promise for accelerated image acquisition while providing consistently high image quality as required for subsequent 3D segmentation workflows^7,8^.

Hence, the present study investigated the clinical applicability of three-dimensional non-selective SSFP imaging using compressed SENSE for low latency endoluminal segmentation combined with multiplanar reformatted imaging planes to facilitate targeted catheter navigation during fully CMR-guided cavo-tricuspid isthmus ablation.

## Methods

### Patient population

Consecutive patients (age > 18 years) with documented isthmus-dependent atrial flutter referred for clinically indicated first-time cavo-tricuspid isthmus (CTI) ablation were enrolled. Patients with general contraindications for CMR imaging (i.e. implanted pacemaker/cardioverter defibrillator), a BMI > 40 kg/m^2^, or a history of congenital heart disease were not considered. In addition, historical ECG documentation of atrial fibrillation episodes or atypical atrial flutter precluded screening for study inclusion. Atrial thrombi were ruled out by transoesophageal echocardiography in all patients presenting at the time with atrial flutter. The study was conducted in accordance with the local institutional review board and the standards of the University of Leipzig ethics committee. Written informed consent was obtained from all patients.

### EP-CMR protocol

Electrophysiological cardiovascular magnetic resonance (EP-CMR) guided intervention was performed on a conventional 1.5 Tesla MR scanner system (Philips Ingenia, Best, The Netherlands) using a 28-element array coil with full in-coil signal digitalization and optical transmission. Patient preparation was done outside the scanner room including the placement of two venous femoral sheaths (10 French) and an arterial radial access (4 French); sedation was initiated and electrical cardioversion was done in patients currently presenting with atrial flutter before transferring the patient to the CMR scanner suite. During the entire procedure, all patients were breathing spontaneously under deep analgosedation (intravenous application of propofol, midazolam and fentanyl as required) and continuously monitored by surface vector ECG, peripheral pulse oximetry, respiratory motion pattern and invasive arterial blood pressure measurements.

**Figure 1:**
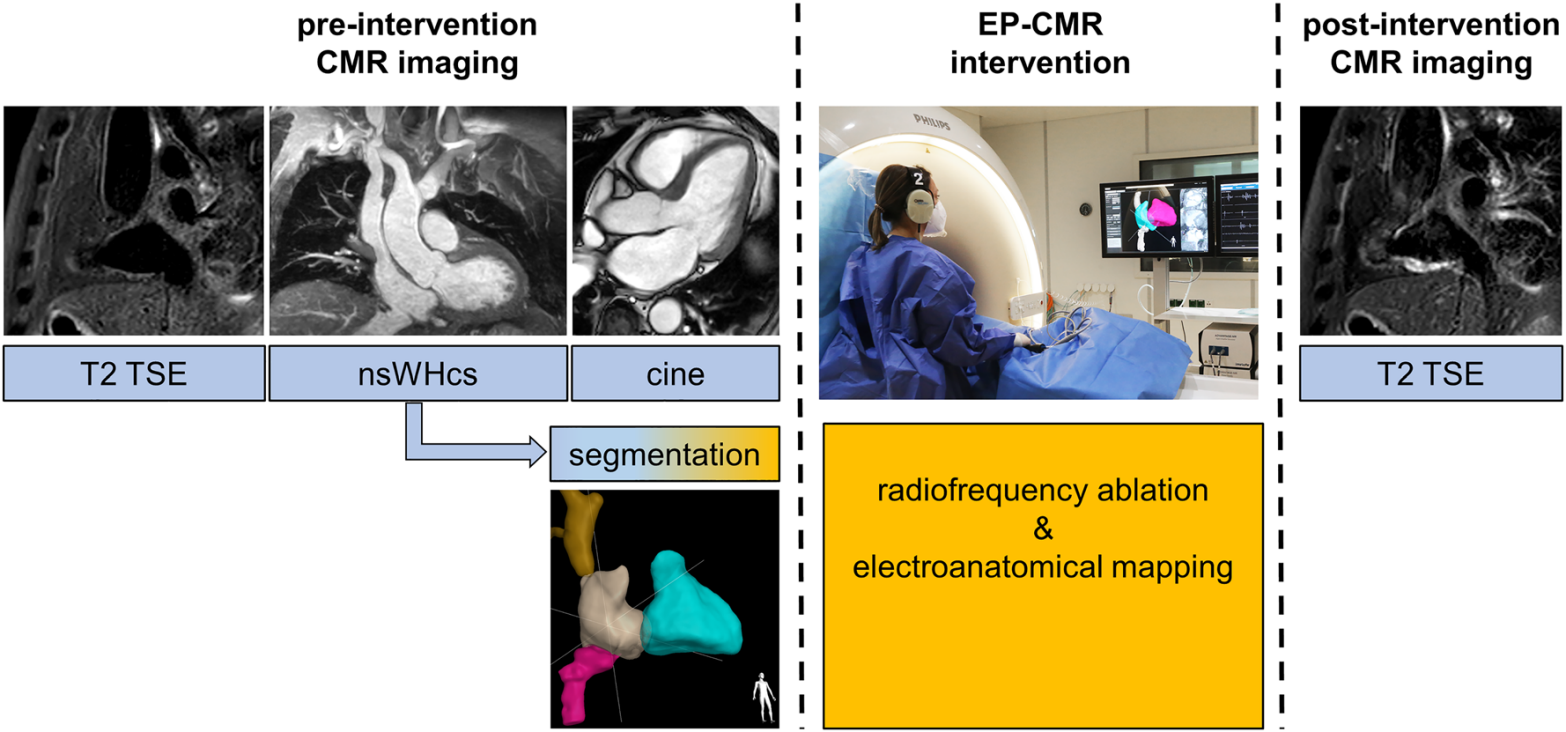
Workflow of fully CMR-guided cavo-tricuspid isthmus ablation. Pre-intervention CMR imaging included T2-weighted imaging of the cavo-tricuspid isthmus followed by a high-resolution 3D whole-heart scan (nsWHcs); while standard cardiac cine image acquisition proceeded, low latency 3D image segmentation was carried out on a networked external workstation by an automated algorithm with the option to apply extended manual segmentation if needed. EP-CMR intervention (i.e. catheter navigation, radiofrequency ablation, activation mapping) relied on real-time active catheter tracking and was based on 3D endoluminal mesh models together with multiplanar reformatted geometries of the coronary sinus or the cavo-tricuspid isthmus, respectively. Post-intervention CMR imaging consisted of repeat T2-weighted imaging in order to confirm RF-induced myocardial edema of the targeted cavo-tricuspid isthmus line.

Pre-interventional CMR imaging consisted of cine images, T2-weighted imaging and a whole-heart sequence for three-dimensional reconstruction of cardiac cavities and large thoracic vessels. CMR-guided radiofrequency ablation of isthmus-dependent atrial flutter was performed using a MR-conditional mapping and ablation irrigation catheter (Vision-MR, Imricor, Burnsville, Minnesota) together with a MR-conditional electrophysiological recording system (Advantage-MR, Imricor, Burnsville, Minnesota) and a standard radiofrequency-pulse generator (IBI-1500T11, St. Jude Medical, St. Paul, Minnesota). The CMR scanner was additionally equipped with an image guidance platform (Interventional MR Suite, iSuite; Philips Hamburg, Germany and Best, The Netherlands) providing full integration with the electrophysiological recording system. Unhindered communication between the staff inside the scanner room and the console room was ensured using an optoacoustic headset system with active noise cancellation (IMROC, Opto-acoustics, Moshav Mazor, Israel). During post-interventional CMR imaging, T2-weighted sequences were repeated in the identical scan geometry. The workflow of the complete EP-CMR procedure has been illustrated in Fig. 1.

### CMR imaging

During deep analgosedation shallow breathing protocols were employed utilizing the acquisition of multiple signal averages or respiratory navigator gating. For pre-interventional imaging, cine images were acquired in all standard cardiac geometries (multiple short-axis views and 4-, 3-, and 2-chamber view); cine imaging generally adhered to current recommendations averaging two signal acquisitions in order to minimize the effect of respiratory variation^9^. Left-ventricular volumes and function were determined from cine images according to standard definitions^10^. For edema detection, a navigator-gated T2-weighted, black-blood turbo-spin echo sequence (TR/TE, > 2000 ms/90 ms; in-plane spatial resolution 1.4 × 1.4 mm^2^; slice thickness, 8 mm) was angulated to the cavo-tricuspid isthmus line with full coverage of the right-sided cardiac cavities (number of slices, 9–12; no gap).

Three-dimensional balanced SSFP imaging was done two minutes after low dose contrast agent application (Dotarem®, Gadoteric acid; 0.05 mmol/kg bodyweight) and was used for three-dimensional surface mesh model reconstruction of cardiac cavities and large thoracic vessels^8^. A large field-of-view with coverage of the entire thorax was acquired in coronal slice orientation with a high and isotropic spatial resolution (reconstructed spatial resolution, 0.9 × 0.9 × 0.9 mm^3^). A T2-preparation prepulse and large flip angle (α = 90°) ensured increased contrast between arterial blood and surrounding tissues. A hard (non-selective) RF block excitation pulse without slice selection gradients provided short repetition and echo times (TR/TE, 2.30 ms/1.15 ms) for low susceptibility to flow artifacts and banding. Compressed SENSE in both phase encoding directions (total acceleration factor 5.0) allowed for short nominal scan durations. The acquisition duration per heartbeat was individually adapted to the diastolic rest period and mainly depended on heart rate (acquisition duration, 100–180 ms). Navigator gating with continuous drift correction (navigator window, 6 mm; scale factor for feet-head correction, 0.45) was used for respiratory motion compensation^11^.

The proposed non-selective balanced SSFP whole heart scan with compressed SENSE (nsWHcs) enabled fully automated segmentation of all cardiac cavities (Heart Segmentation; IntelliSpace Portal 12.0, Philips, Best, The Netherlands). The software delivered three-dimensional reconstructions of the right atrium, right ventricle, left atrium, left ventricle, left-ventricular myocardium, and ascending aorta; the duration of fully automated nsWHcs segmentation was recorded. The results of automated segmentation were immediately checked by a cardiological CMR imaging expert (I.P., > 25 years of experience, SCMR and ESC level 3 certified) with regard to correct endoluminal contour delineation of the targeted right-sided cavities (i.e. right atrium, right atrial appendage, coronary sinus ostium, tricuspid annulus, and right ventricle), and manually corrected if necessary. Subsequently, extended segmentation of the inferior and superior vena cava and the course of the coronary sinus were manually added (3D Segmentation, CT Viewer; IntelliSpace Portal 12.0, Philips, Best, The Netherlands) and total manual processing time was recorded. All segmented right-sided cavities were then converted to surface mesh models and loaded into the display of the interventional suite for subsequent three-dimensional catheter navigation.

**Figure2:**
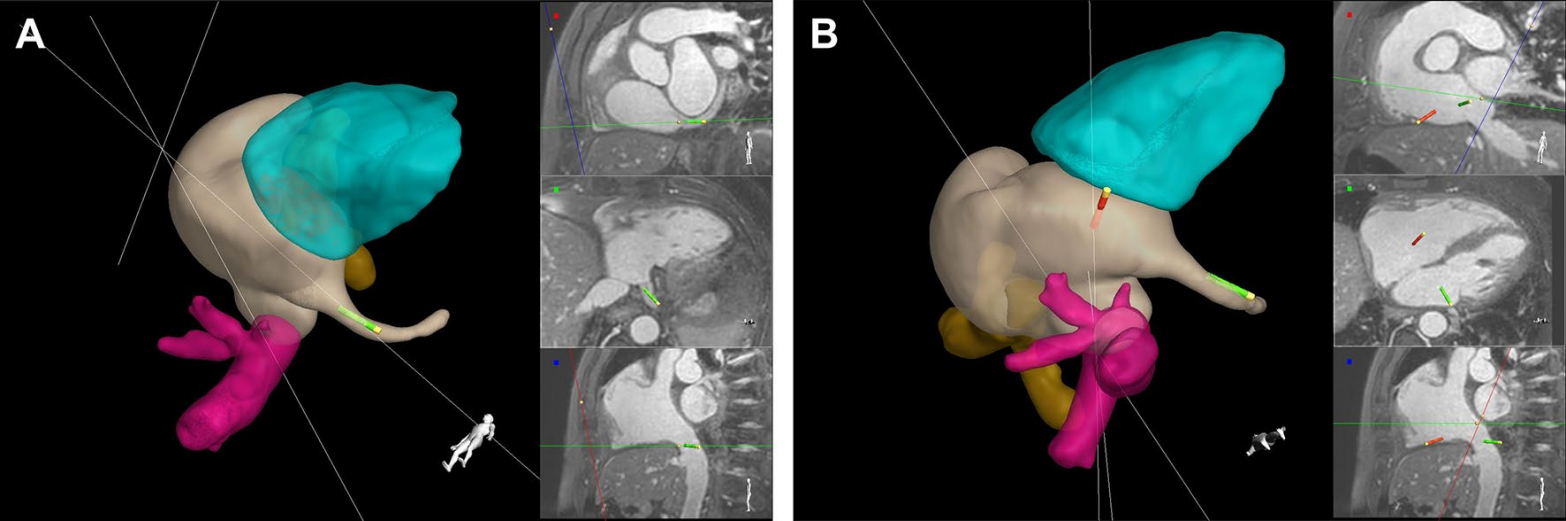
Standardized displays of 3D endoluminal models and multi-planar reformatted geometries of nsWHcs during EP-CMR intervention. Endoluminal 3D models were permanently displayed in the large left-sided viewport and the CMR operator provided positional adjustment according to procedural need. Multiplanar reformatted geometries of nsWHcs with simultaneous real-time display of catheter tip position were given in the three right-sided viewports. (**A**) Monitor display during insertion and positioning of the reference catheter. Left, 3D endoluminal surface mesh models with the green catheter tip of the reference catheter placed in the coronary sinus. Right-top, multiplanar reformatted basal short axis orientation showing the full course of the coronary sinus; right-center, transversal geometry illustrating the angle between inferior vena cava and coronary sinus ostium; right-bottom, sagittal geometry providing full length view of the inferior vena cava during initial catheter push-up. After successful placement of the reference catheter within the coronary sinus, the viewing display was switched to the predefined isthmus geometry shown in figure (**B**). (**B**) Monitor display during point-by-point ablation of the cavo-tricuspid isthmus and activation mapping. Left, 3D endoluminal surface mesh models with the reference catheter positioned in the coronary sinus (green catheter tip) while the ablation catheter (red catheter tip) was placed at the tricuspid annulus. Right-top, multiplanar reformatted cavo-tricuspid isthmus geometry; right-center, 4-chamber orientation providing an orthogonal view of the targeted cavo-tricuspid isthmus line; right-bottom, sagittal geometry with full length visualization of the inferior vena cava.

**Figure 3:**
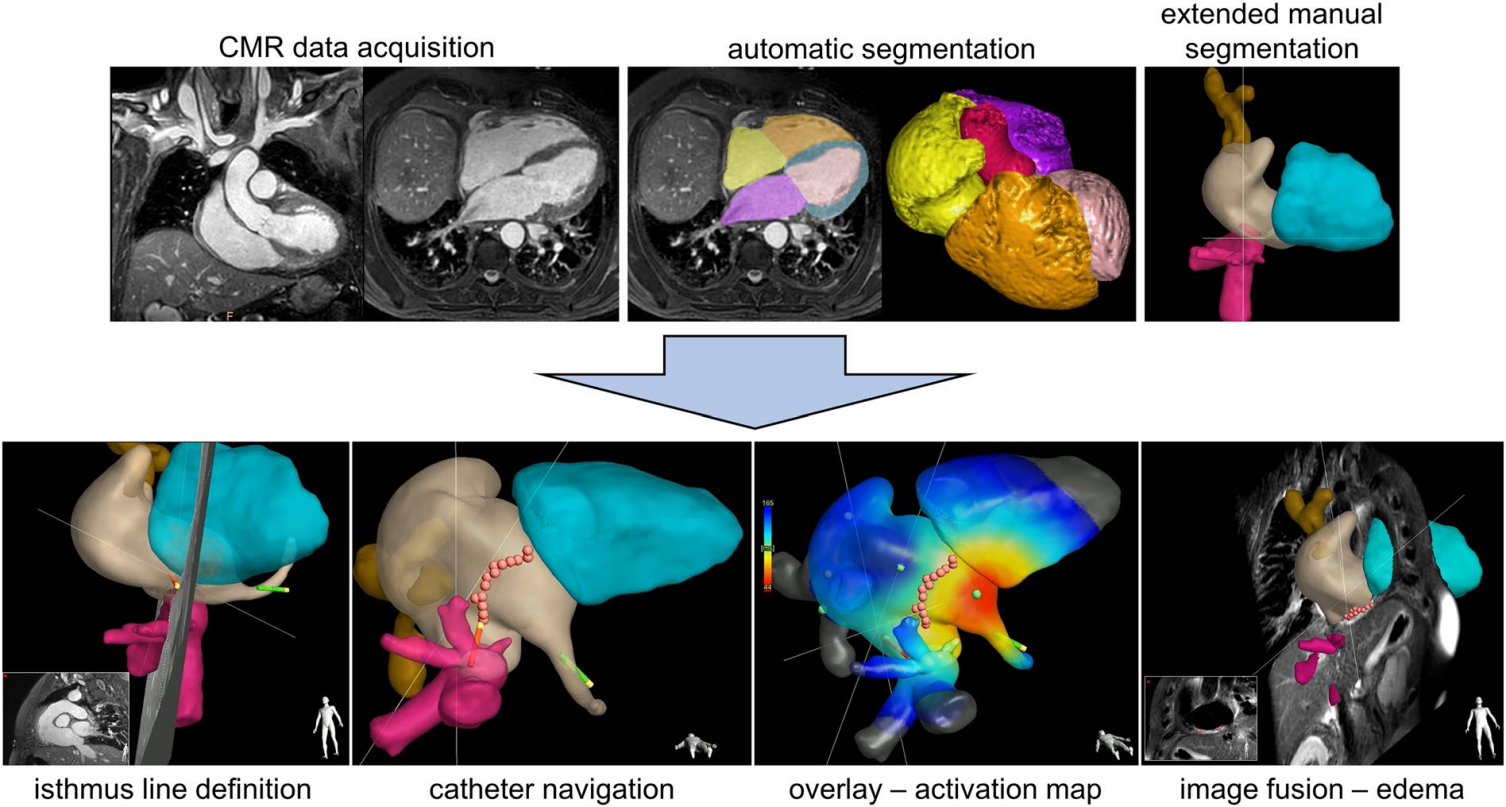
Image processing of nsWHcs and information gain for EP-CMR interventions. Upper row: During pre-intervention CMR imaging, nsWHcs image data were acquired with subsequent automated segmentation of the cardiac cavities in order to ensure low latency image reconstruction. The automatic segmentation results were checked and manually extended if needed to improve anatomical orientation for the interventionalist/electrophysiologist. The final set of 3D mesh models was employed for 3D EP-CMR interventional guidance. Bottom row: During EP-CMR intervention, 3D endoluminal surface mesh models derived from nsWHcs ensured precise catheter navigation and could be combined with any multiplanar reformatted geometry for visualization of surrounding tissue (e.g. predefined cavo-tricuspid isthmus line), fused with any other imaging sequence (e.g. edema imaging), and overlaid with any additional catheter-related information at the tip position (e.g. activation time measurements). Thus, nsWHcs can be considered the core imaging component of 3D EP-CMR interventional procedures.

Multi-planar reformatting of nsWHcs provided standardized geometries of the coronary sinus and cavo-tricuspid isthmus (see Fig. 2). In-plane visualization of coronary sinus comprised of a basal short axis geometry at the level of the mitral annulus (= CS ostium and full-length depiction), an orthogonal view in transversal orientation at the level of the CS ostium (= definition of the angle between IVC and CS) and a sagittal view of the IVC in full length (= definition of IVC course and its confluence angle with RA). In addition, cavo-tricuspid isthmus geometry was prescribed in consensus between the cardiological CMR imaging expert and the electrophysiologist (= targeted line for point-by-point radiofrequency ablation) as follows: the standard viewing display comprised of an in-plane view of cavo-tricuspid isthmus length, an orthogonal view in transversal orientation (= resembling a slightly angulated 4-chamber orientation), and a sagittal view of the IVC course to uncover eustachian valve or IVC/right atrial pouch configuration (see Fig. 2B). At the discretion of the electrophysiologist, these multiplanar reformatted geometries were combined with the segmented endoluminal 3D models at any time (see Fig. 3). Right atrial volume was derived from the segmented nsWHcs dataset and cavo-tricuspid isthmus length was measured on the predefined multiplanar reformatted geometry. Preprocedural imaging duration was defined as the time period between start of first CMR scan to insertion of first catheter.

### CMR-guided radiofrequency ablation

During the entire EP-CMR intervention, active catheter tracking provided real-time catheter visualization and enabled precise catheter navigation within the three-dimensional surface mesh models^3,12^. The first catheter was used for coronary sinus cannulation and served as the reference catheter. The second catheter was for radiofrequency ablation and was initially placed at the tricuspid annulus targeting the predefined cavo-tricuspid isthmus line. Catheter contact to the atrial wall was ensured on corresponding multiplanar reformatted views of nsWHcs imaging (see Fig. 2B). A point-by-point ablation line was created by gradual pull-back of the ablation catheter along the isthmus line with the following parameter settings per ablation point: duration, max. 60 s; power, max. 65 W; temperature, max. 40 °C; and irrigation flow, 17 ml/min. An ablation point marker was set at the position of the catheter tip after at least 30 s of continuous RF application; the total number of ablation points was recorded.

A successful bidirectional isthmus block was verified by postablation mapping with pacing maneuvers from the reference catheter at the proximal coronary sinus. Colour-coded activation maps were derived from catheter-based time delay measurements taken between mapping and reference electrograms. The lower limit of the colour scale was defined by the shortest activation time on the coronary sinus side of the ablation line (A1); the longest activation time defined the upper limit of the colour scale and was measured on the opposite side of the ablation line (A2). A conduction time difference > 100 ms across the ablation line was considered indicative of complete cavo-tricuspid isthmus block. In general, multiple mapping points were taken along the circumference of the tricuspid annulus to determine activity propagation and to further substantiate the complete isthmus block (see Fig. 3). EP-CMR procedure duration was defined as the time period between insertion of the first catheter to removal of the last catheter from the patient.

### Statistical analysis

All analyses were done using SPSS (version 21, IBM Corporation, Armonk, NY). Continuous variables were stated as mean ± standard deviation if normally distributed; numbers and ratios were used to describe categorical variables. Pearson correlation was used to evaluate the linear relationship between continuous variables. A two-tailed *p* value < 0.05 was considered statistically significant.

### Ethics approval and consent to participate

Ethics approval was obtained by the local ethics committee of the faculty of medicine of the University of Leipzig. For each subject written informed consent was obtained.

## Results

### Patient population

Fourty-two study participants with isthmus-dependent right atrial flutter were included. Patients predominantly exhibited sinus rhythm (27/42, 64%) while all others underwent successful electrical cardioversion directly before CMR imaging (15/42, 36%). Detailed patient characteristics are given in Table 1.

**Table 1:** Patient characteristics and CMR imaging parameters.

	All patients (n = 42)
Age, years	65 ± 12
Gender, male	39 (93)
BMI, kg/m^2^	29.0 ± 3.7
Hypertension	37 (88)
Diabetes	11 (26)
Known CAD	9 (21)
Heart rate, bpm	67 ± 10
Systolic blood pressure, mmHg	123 ± 21
LVEDV, ml	150 ± 30
LVEF, %	60 ± 6
LA, cm^2^	27 ± 5
RA, cm^2^	25 ± 5

Values are mean ± SD or n (%).

*BMI* body mass index, *CAD* coronary artery disease, *LV* left-ventricular, *EDV* end-diastolic volume, *EF* ejection fraction, *LA* left atrium, *RA* right atrium.

### Non-selective whole heart imaging using compressed SENSE (nsWHcs)

**Figure 4:**
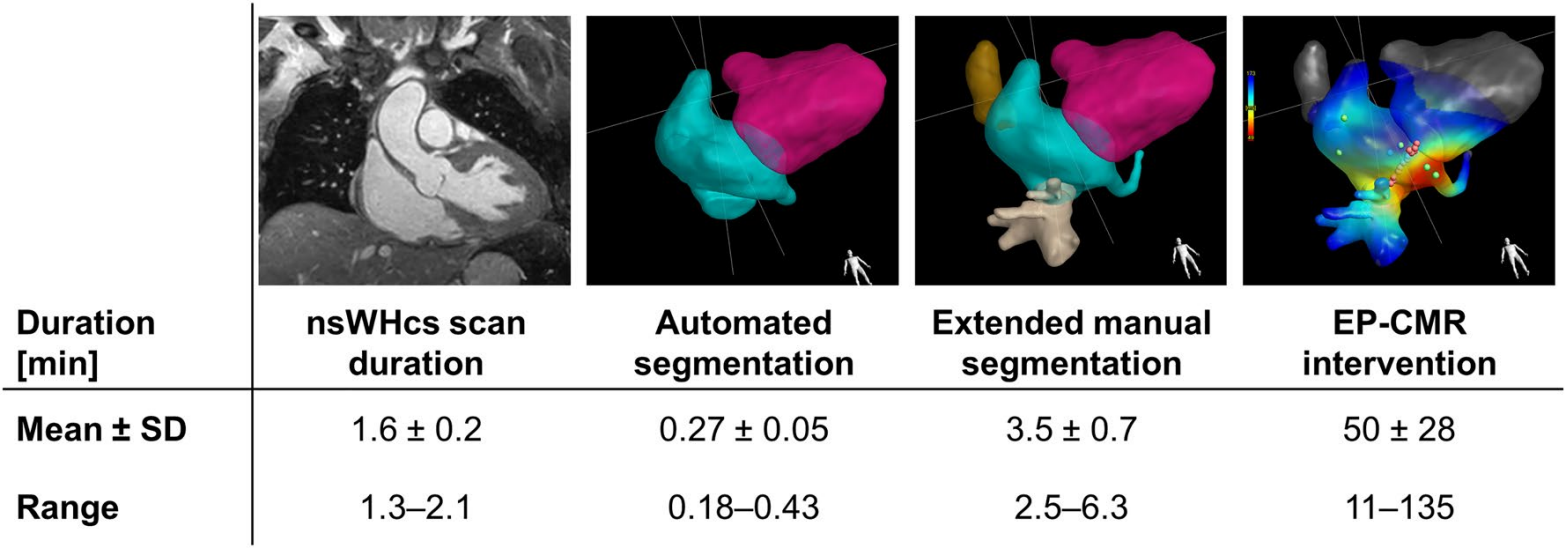
Processing time of nsWHcs and EP-CMR procedure duration. Mean duration of the procedural components of fully CMR-guided radiofrequency ablation of isthmus-dependent right atrial flutter. *SD* standard deviation, *CMR* cardiovascular magnetic resonance, *EP-CMR* CMR-guided electrophysiological procedure.

The pre-interventional CMR imaging protocol was completed successfully in all patients (42/42; 100%). Mean nominal scan duration of nsWHcs was 98 ± 10 s (range 79‒126 s; see Fig. 4). Fully automated segmentation of nsWHcs took 16 ± 3 s and delivered appropriate three-dimensional endoluminal contour delineation of the right-sided cardiac cavities in all but two patient datasets which required manual correction of coronary sinus ostium geometry (2/42; 5%; see Table 2). Mean RA volume was 148 ± 48 ml (range 72‒236 ml). Multi-planar reformatting of nsWHcs enabled adequate coronary sinus visualization (> 5 cm vessel length) as well as pre-interventional geometry definition of the cavo-tricuspid isthmus line in all patients (42/42; 100%). Mean cavo-tricuspid isthmus length was 31 ± 6 mm (range 19‒40 mm) and cavo-tricuspid isthmus length correlated with RA volume (r = 0.48, *p* = 0.001).

**Table 2:** Fully automated segmentation of nsWHcs: successful endoluminal delineation of right-sided cardiac cavities.

	All patients (n = 42)
Right atrium	42 (100)
Right atrial appendage	42 (100)
Coronary sinus ostium	40 (95)
Tricuspid annulus	42 (100)
Right ventricle	42 (100)

Values are given as n (%).

### EP-CMR intervention

EP-CMR procedure parameters are given in detail in Table 3.

**Table 3:** EP-CMR procedure parameters.

Procedure parameter	All patients (n = 42)
CS cannulation success rate	42 (100%)
Number of ablation points	18 ± 11
Range	[6, 43]
Alignment with predefined isthmus line	26 (62%)
Deviation of > 1 marker width	16 (38%)
Maximum conduction time difference, ms	136 ± 28
Range, ms	[101, 225]
Preprocedural imaging duration, min	19 ± 3
Range, min	[14, 26]

Values are mean ± SD or n (%), range values are given in square brackets. CS indicates coronary sinus.

In one patient, atrial fibrillation occurred during the intervention and persisted until completion of the point-by-point ablation line; postablation myocardial edema along the entire cavo-tricuspid isthmus line was documented but the electrical endpoint could not be verified by activation mapping. After the patient exited the CMR scanner suite, successful electrical cardioversion was performed and a stable sinus rhythm could be documented for the next three months after the EP-CMR procedure. In the remaining 41 patients (41/42; 98%) activation mapping confirmed complete bidirectional isthmus block (i.e. a maximum conduction time difference between both sides of the ablation line > 100 ms, see Table 3). Conduction time difference did not correlate with RA volume (r = 0.113, *p* = 0.482) or isthmus length (r = 0.167, *p* = 0.297), respectively. Similarly, isthmus length did not correlate with the number of ablation points (r = 0.012, *p* = 0.94) or interventional procedure duration (r = 0.146, *p* = 0.36), respectively.

## Discussion

The main findings of the current study were as follows: the proposed 3D whole heart imaging approach using a non-selective balanced SSFP sequence with compressed SENSE (1) resulted in a short nominal scan duration (98 ± 10 s), (2) facilitated fully automated 3D segmentation of cardiac cavities with a high success rate (95‒100%), (3) enabled combined visualization of 3D endoluminal mesh models with any multiplanar reformatted geometry of surrounding tissue or any other CMR imaging sequence (e.g. edema imaging), (4) represented the basis for precise catheter navigation during anatomically-defined electrophysiological ablation procedures, and (5) could be used for parametric overlay with any catheter-tip measurements (e.g. activation time).

Previous EP-CMR studies made use of a conventional, ECG-triggered, navigator-gated 3D SSFP sequence^3,13^. However, 3D SSFP sequences exhibited a few principal disadvantages, i.e. relatively long scan durations (approximately 7–12 min), a high susceptibility to artifacts caused by turbulent flow and metallic implants provoking magnetic field inhomogeneities, and a generally reduced image quality in patients with an irregular heartbeat. In the present study, these shortcomings were addressed by using a non-selective radiofrequency excitation (= reduced endoluminal signal loss/image artifacts of SSFP sequences) in combination with compressed SENSE (= considerable speed-up of image data acquisition)^7,14,15^. The use of a non-selective radiofrequency excitation very effectively minimized the susceptibility of SSFP sequences to B0 magnetic field inhomogeneities and out of slice contributions (thereby preventing signal loss and image artifacts), but required a large field-of-view with coverage of the entire thorax resulting in unacceptably long scan durations. Only in combination with the speed-up provided by recently introduced compressed sensing techniques, rapid and complete 3D whole thorax imaging became clinically useful and resulted in short effective scan durations, typically in the range of 2–3 min.

Non-contrast SSFP acquisitions are generally prone to signal voids or banding artifacts: our group demonstrated in a previous study, that endoluminal signal variations can be overcome with nsWHcs data acquisition during the postcontrast pseudo-equilibrium/equilibrium phase of Gadolinium-containing contrast agents^8^. The uniformly high endovascular luminal signal achieved with postcontrast nsWHcs together with fully automatic segmentation translated into a significant reduction in preprocedural imaging duration (30 ± 5 min for conventional SSFP whole heart versus 19 ± 3 min for nsWHcs)^3^. Overall procedure duration (i.e. preprocedural imaging plus EP-CMR procedure duration) ranged from 29 to 153 min and was, thus, comparable to prior studies on fluoroscopy-guided atrial flutter ablation reporting procedure durations ranging from 31 to 180 min^16^.

Previous investigators described a close relationship of fluoroscopy-guided atrial flutter ablation procedure duration with isthmus length and right atrial size/the number of ablation points^16^. In our study, however, we did not find a correlation between isthmus length or right atrial dimensions and interventional procedure duration. One may hypothesize, that direct 3D visualization of atrial anatomy/geometry during the ablation procedure together with a correctly predefined isthmus line may abolish the relationship. However, this needs to be addressed in more detail in future research.

Perspectively, the 3D nsWHcs scan has a broad scope of application possibilities and is extremely easy to incorporate into routine clinical practice: since nsWHcs data acquisition is preferably performed during the pseudo-equilibrium phase of Gadolinium-containing extracellular contrast agents (i.e. 1–4 min after contrast agent application), nsWHcs scans can be employed to eliminate “dead time” in the vast majority of current standard cardiovascular MR protocols.

The fully CMR-guided electrophysiological procedure benefitted from the precise, 3D endoluminal anatomical roadmap without the need for laborious and error prone co-registration together with the permanent availability of any desired additional image information on extraluminal anatomy or tissue properties. Clearly, high quality (i.e. high isotropic spatial resolution) and artifact-free image data acquisition was mandatory for rapid and robust 3D endoluminal segmentation or on-the-fly multiplanar reformatting. Since pre-interventional imaging was carried out in the sedated patient, the demand for fast image data acquisition and low latency 3D image segmentation was eminently important. Nominal scan duration of nsWHcs was consistently less than two minutes (98 ± 10 s) and isotropic voxel size with submillimeter spatial resolution allowed for high-quality multiplanar reformatting of coronary sinus and cavo-tricuspid isthmus geometries in all patients. These findings corroborated previous study results, introducing nsWHcs for diagnostic assessment of children and adults with congenital heart disease and reporting an overall homogeneous signal intensity distribution and excellent image quality^8^.

For conventional electrophysiology procedures under fluoroscopic guidance, pre-interventional CMR or CT imaging is usually done several hours or days before the intervention and thus, segmentation time is generally of lesser concern. For fully CMR-guided interventional procedures, the deeply sedated patient remains in the same position during the time period from pre-interventional imaging to the ablation procedure followed by post-interventional imaging. Hence, readily available 3D endoluminal segmentation results are key to initiate and proceed safely throughout the ablation procedure. Notably, the proposed automated segmentation approach needed an overall short computation time (16 ± 3 s) and resulted in flawless 3D models of the right atrium and right ventricle with exact tricuspid annulus depiction in all patients. Extended manual segmentation could easily be added as required and in our study was routinely done for SVC, IVC and the course of the coronary sinus in order to supply the EP-interventionalist with detailed anatomy. Perspectively, further development of artificial intelligence-based algorithms may render any additional manual segmentation of cavitary/vascular structures unnecessary.

Side-by-side display of endoluminal 3D mesh models and multiplanar reformatted geometries of nsWHcs facilitated precise catheter navigation and allowed for permanent verification of direct catheter contact with the ablation target/right-atrial wall. Display and viewport arrangements of reformatted geometries may vary according to the preferences of the electrophysiologist but we favored CMR imaging geometries closely resembling well established fluoroscopic angulations thereby increasing familiarity of the interventionalist with the full-view anatomical information content^17^. For the current study, two standardized sets of reformatted geometries were displayed for the coronary sinus and the cavo-tricuspid isthmus, respectively, and provided the electrophysiologist with a detailed roadmap during the entire interventional procedure (see Fig. 2).

In general, the introduction of fully CMR-guided ablation procedures is driven by the potential to fully exploit the unrivaled advantages of CMR imaging regarding 3D image data acquisition of myocardial tissue characteristics. In particular substrate-guided ventricular ablation procedures mandate the distinct visualization of potential arrhythmogenic targets. In patients with ventricular rhythm disorders, pre-interventional myocardial texture characterization by CMR imaging has already proved advantageous^18,19^. Reportedly, image fusion of tissue characterization sequences (e.g. edema imaging, late gadolinium enhancement) and endoluminal 3D surface mesh models to obtain key anatomical information was considered beneficial for substrate-based ablation procedures^20^. However, these studies mainly relied on CMR late gadolinium enhancement imaging for myocardial substrate definition together with CT angiography for anatomy depiction^20,21^. Taking advantage of the proposed nsWHcs imaging approach may render additional CT examinations and error prone landmark registration of CT-based mesh models used for subsequent 3D catheter navigation unnecessary. Prior studies demonstrated that CMR-aided VT substrate ablation was associated with shortened procedure durations and enhanced ventricular arrhythmia free survival^19,20^. Thus, substrate-based ablation of ventricular arrhythmia under full CMR-guidance within the CMR environment would unleash the full power of CMR imaging and constitute a real game-changer for electrophysiological treatment. The present study showed that CMR imaging is ready to advance from anatomically defined to fully substrate-dependent CMR-guided interventions when the technical challenges of re-engineering all necessary catheter devices for use in the MR environment are overcome.

## Conclusions

The proposed nsWHcs approach allowed for fast image acquisition and low latency 3D segmentation of endoluminal cavities during pre-interventional imaging for fully CMR-guided isthmus-dependent right atrial flutter ablation. Combined visualization of 3D surface mesh models and multiplanar reformatted geometries ensured precise real-time catheter navigation throughout the anatomically defined electrophysiological procedure. In addition, 3D endoluminal models could be overlaid with additional sequences for tissue characterization (e.g. edema imaging) or catheter-related measurements (e.g. activation time). Thus, nsWHcs constituted the key imaging component for fully CMR-guided electrophysiological interventions. Perspectively, the full potential of EP-CMR intervention will be unveiled in substrate-guided ventricular ablation procedures that will take advantage of the inherent CMR-tissue characterization capabilities.

### Supplementary Information

Supplementary Legends.

Supplementary Video 1.

## Data Availability

The authors confirm that the data supporting the findings of this study are available within the article and its Supplementary materials.

## Abbreviations

CMR: Cardiovascular magnetic resonance
CS: Coronary sinus
CTI: Cavo-tricuspid isthmus
EP-CMR: Electrophysiological cardiovascular magnetic resonance
IVC: Inferior vena cava
LA: Left atrium
nsWHcs: Non-selective whole heart SSFP imaging using compressed SENSE
RA: Right atrium
RF: Radiofrequency
SENSE: Sensitivity encoding
SSFP: Steady-state free precession
