# Supplementary material for: Electrophysiological cardiovascular MR: procedure-ready mesh model generation for interventional guidance based on non-selective excitation compressed sensing whole heart imaging
Source: Sci Rep. 2024 Apr 18;14:8974. doi: 10.1038/s41598-024-59230-0 (PMC11026457; doi:10.1038/s41598-024-59230-0)
Supplement: Supplementary file 1 — Supplementary Legends. [file 41598_2024_59230_MOESM1_ESM.docx]

# Supplementary Material

## Video 1 (.MP4 file)

**Fully CMR-guided cavo-tricuspid isthmus ablation (EP-CMR) in typical right atrial flutter.**

Recording of a complete EP-CMR procedure for cavo-tricuspid isthmus ablation in typical right atrial flutter: (1) Coronary sinus cannulation with reference catheter (green catheter tip) using real-time active catheter tracking (viewport A: mesh surface models of right atrium and coronary sinus ostium, aqua; right ventricle, magenta; superior/inferior vena cava, goldenrod/beige) with simultaneous display of catheter position on standardized coronary sinus geometries (viewports B-D). (2) subsequently, the radiofrequency ablation catheter (red catheter tip) was positioned at the tricuspid annulus and (3) point-by-point radiofrequency ablation of the cavo-tricuspid isthmus following a predefined anatomical line was carried out (red dots, ablation sites); (4) finally, activation mapping confirmed complete bidirectional isthmus block (green dots, activation time mapping sites; trans-isthmus conduction time, 132 ms).

Total procedure duration ("first catheter in - last catheter out") amounted to 11 min; for ease of viewing, "burns" (i.e. 60 seconds durations of radiofrequency application with the catheter remaining in the position indicated by the red ablation dots) were cut down to 10 seconds.
